# Aligning Noninferiority Assumptions and Decision Rules in a Protocol for a Study on Adjunctive Acupuncture for Late-Life Depression

**DOI:** 10.2196/92775

**Published:** 2026-05-01

**Authors:** Kenjiro Shiraishi

**Affiliations:** 1 Tanashi Kitaguchi Acupuncture and Moxa Clinic, Nozaki Building 301 2-9-6 Tanashi-cho, Nishi-Tokyo, Tokyo, Japan, 81 0424974586

**Keywords:** depression, mild to moderate depression in older people, acupuncture, randomized controlled trial, protocol

Fu and colleagues’ [1] protocol comparing citalopram plus acupuncture versus citalopram alone for mild to moderate major depressive disorder in older adults addresses an important clinical question. Because the trial is positioned as a noninferiority study, interpretability depends on clear alignment between (1) the primary end point definition, (2) the assumptions used for planning, and (3) prespecified decision rules for inference [2].

In nonblinded designs with unequal treatment contact time, response-based binary end points may be sensitive to nonspecific effects (eg, expectancy and attention), making end point–consistent assumptions and transparent inferential conventions especially important for interpreting noninferiority conclusions [3].

The protocol defines the primary end point as 17-item Hamilton Depression Rating Scale (HAMD-17) response at week 12 (≥50% reduction from baseline) [1]. In the Sample Size Calculation section [1], the assumed control-group rate (about 30%) is justified by reference to the Sequenced Treatment Alternatives to Relieve Depression (STAR*D) study [4]. However, commonly cited STAR*D estimates often refer to citalopram remission rates at level 1 (approximately 30%‐33%, depending on the instrument), which are conceptually and operationally distinct from the response [4]. If planning assumptions draw on remission-based estimates but the primary end point is the response, the planning rationale may not map cleanly onto the stated end point, and sensitivity to plausible response-rate assumptions becomes relevant for interpreting power and margin adequacy.

Readers may also look for a clearly prespecified noninferiority decision rule for the binary end point (eg, effect measure, CI approach, and the criterion for noninferiority relative to Δ = −15%), consistent with CONSORT (Consolidated Standards of Reporting Trials) guidance, and for consistency between the alpha used in sample size planning and the analysis description [2].

Because conclusions can be sensitive to analysis populations and missing data, clarity on intention-to-treat versus per-protocol roles and missing-data handling can further support interpretation [2,3].

These considerations may help readers place the protocol’s design choices in a broader context when interpreting results from noninferiority trials of complex adjunctive interventions.

## Abbreviations

CONSORT: Consolidated Standards of Reporting Trials
HAMD-17: 17-item Hamilton Depression Rating Scale
STAR*D: Sequenced Treatment Alternatives to Relieve Depression
